# Diagnostic Performance of Noninvasive Coronary Computed Tomography Angiography-Derived FFR for Coronary Lesion-Specific Ischemia Based on Deep Learning Analysis

**DOI:** 10.31083/j.rcm2501020

**Published:** 2024-01-10

**Authors:** Haoyu Wu, Lei Liang, Fuyu Qiu, Wenqi Han, Zheng Yang, Jie Qi, Jizhao Deng, Yida Tang, Xiling Shou, Haichao Chen

**Affiliations:** ^1^Department of Cardiology, Shaanxi Provincial People’s Hospital, 710068 Xi’an, Shaanxi, China; ^2^Department of Cardiology, Sir Run Run Shaw Hospital, Zhejiang University School of Medicine, 310018 Hangzhou, Zhejiang, China; ^3^Key Laboratory of Cardiovascular Intervention and Regenerative Medicine of Zhejiang, 310018 Hangzhou, Zhejiang, China; ^4^Department of Cardiovascular Medicine, Peking University Third Hospital, 100191 Beijing, China

**Keywords:** coronary artery disease, coronary lesion-specific ischemia, fractional flow reserve (FFR), computed tomography angiography-derived FFR (CT-FFR), coronary computed tomographic angiography, deep learning analysis

## Abstract

**Background:**

The noninvasive computed tomography angiography–derived fractional flow reserve (CT-FFR) can be used to diagnose coronary ischemia. With advancements in associated software, the diagnostic capability of CT-FFR may have evolved. This study evaluates the effectiveness of a novel deep learning-based software in predicting coronary ischemia through CT-FFR.

**Methods:**

In this prospective study, 138 subjects with suspected or confirmed coronary artery disease were assessed. Following indication of 30%–90% stenosis on coronary computed tomography (CT) angiography, participants underwent invasive coronary angiography and fractional flow reserve (FFR) measurement. The diagnostic performance of the CT-FFR was determined using the FFR as the reference standard.

**Results:**

With a threshold of 0.80, the CT-FFR displayed an impressive diagnostic accuracy, sensitivity, specificity, area under the receiver operating characteristic curve (AUC), positive predictive value (PPV), and negative predictive value (NPV) of 97.1%, 96.2%, 97.7%, 0.98, 96.2%, and 97.7%, respectively. At a 0.75 threshold, the CT-FFR showed a diagnostic accuracy, sensitivity, specificity, AUC, PPV, and NPV of 84.1%, 78.8%, 85.7%, 0.95, 63.4%, and 92.8%, respectively. The Bland–Altman analysis revealed a direct correlation between the CT-FFR and FFR (*p* < 0.001), without systematic differences (*p* = 0.085).

**Conclusions:**

The CT-FFR, empowered by novel deep learning software, demonstrates a strong correlation with the FFR, offering high clinical diagnostic accuracy for coronary ischemia. The results underline the potential of modern computational approaches in enhancing noninvasive coronary assessment.

## 1. Introduction

While invasive coronary angiography (ICA) provides limited anatomical information on the coronary artery, the results often form the basis for the decision to perform percutaneous coronary intervention (PCI) [1]. This reliance on ICA results in undesired outcomes, such as unnecessary PCI for functionally insignificant lesions or improper delays in PCI for functionally significant lesions [1]. An alternative method, the fractional flow reserve (FFR), serves as a hemodynamic correlation criterion that enhances the benefits of revascularization, improves event-free survival, and reduces health costs [2]. Despite its advantages, the invasive nature of FFR measurement, its need for expensive equipment, and the potential complications it may cause to the coronary artery limit its routine use in clinical practice.

For patients with low or moderate risk coronary artery disease (CAD), noninvasive tests such as the anatomy-based coronary computed tomography angiography (CCTA) can be attempted prior to more invasive testing [3, 4]. While CCTA is considered the first-line approach [4], with strengths including a high sensitivity (87%–99%) and moderate specificity (61%–83%) [5], the relatively high false-positive rate may lead to an increase in the need for ICA. More concerning is CCTA’s inability to assess the physiological function of the coronary artery based on the severity of coronary anatomical stenosis alone.

A promising solution to these limitations is the noninvasive computed tomography angiography–derived FFR (CT-FFR) [6]. This method can assess lesion-specific ischemia via computational fluid dynamics (CFD) without requiring changes to the CCTA data collection protocol, additional imaging, or drugs [6]. Impressively, CT-FFR has demonstrated an overall accuracy of 85% sensitivity and 82% specificity in pinpointing lesion-specific ischemia [6]. To streamline the integration of CT-FFR into clinical workflows and improve diagnostic accuracy, new software and algorithms have been created. These innovations facilitate cost-effective CT-FFR analyses on a standard workstation, eliminating the need for unnecessary ICA.

Machine learning-based flow assessments using artificial intelligence algorithms have recently been introduced to perform CT-FFR analysis. Coenen *et al*. [7] and Qiao *et al*. [8] suggested a supervised learning approach that involved training with diverse features from different anatomies and degrees of CAD, utilizing reduced-order CFD to compute CT-FFR values. In this prospective study, we assessed the diagnostic characteristics of CT-FFR by employing new deep learning software specifically designed for coronary lesion-specific ischemia analysis. This novel software package consists of two components: the Coronary Scope, a deep learning tool for evaluating the physiological function of the coronary artery, and the Compute Unified Device Architecture (CUDA) accelerated CFD software, tailored for analyzing incompressible fluid flow equations. These clinical experiments were conducted to evaluate the ability of the CT-FFR to identify coronary ischemia at FFR thresholds of 0.80 and 0.75, providing insights into its effectiveness and potential applications in CAD diagnosis.

## 2. Materials and Methods

### 2.1 Study Design and Study Population

This prospective trial evaluated the diagnostic characteristics of the CT-FFR with a novel software research prototype (coronary artery physiological function assessment software: Coronary Scope V1.0, Shenzhen Yueying Technology Co., Ltd., Shenzhen, China) to diagnose lesion-specific ischemia in subjects with suspected or known CAD. The CT-FFR was evaluated for stenosis in one target vessel per patient. This study protocol was approved by the Institutional Audit Committee of Shaanxi Provincial People’s Hospital. Informed written consent was obtained from all participants.

**Fig. 1.**
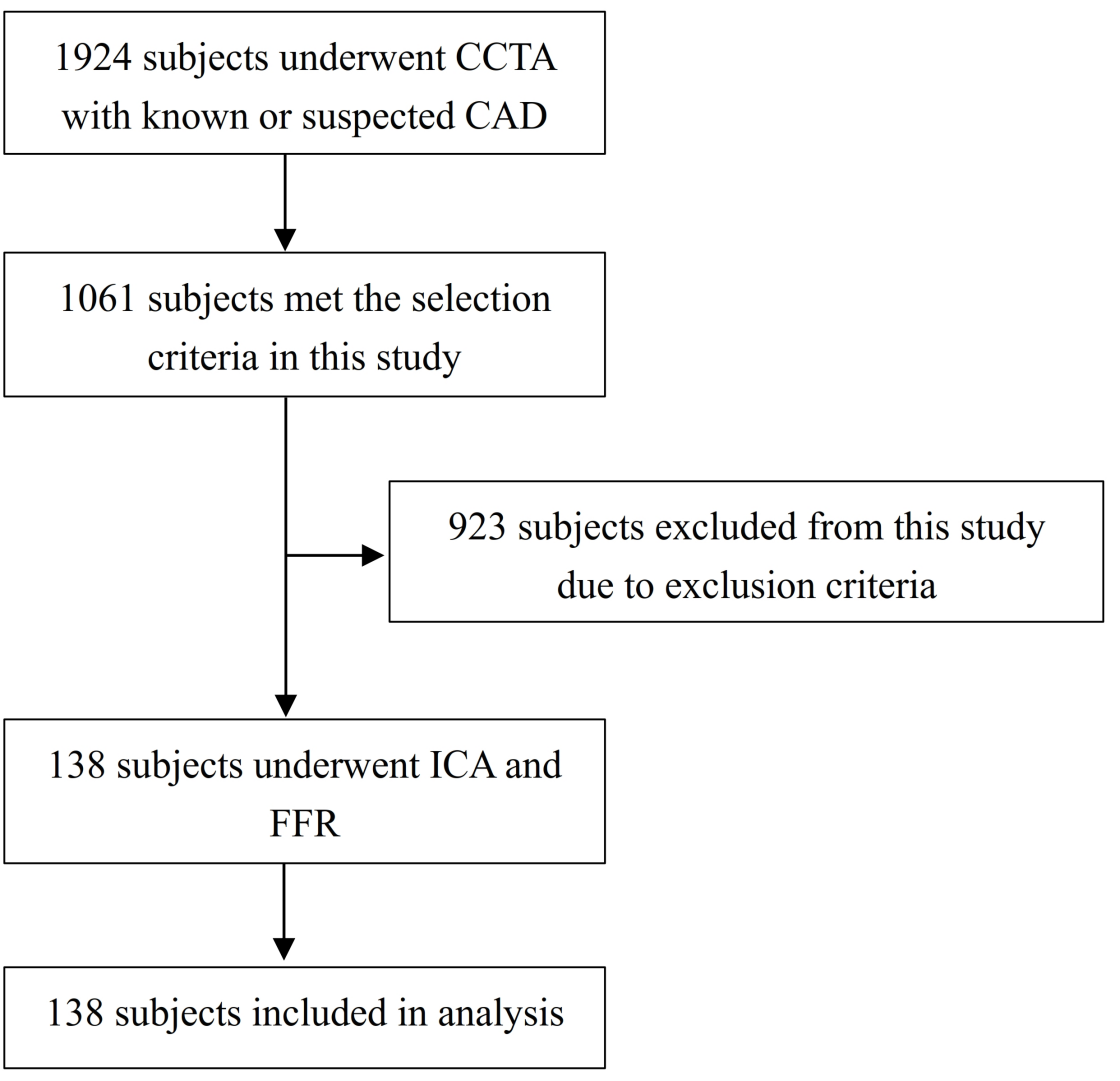
**Flowchart of patient recruitment**. CAD, coronary artery disease; CCTA, coronary computed tomography angiography; FFR, fractional flow reserve; ICA, invasive coronary angiography.

The study included patients with known or suspected CAD who underwent ICA and FFR measurement after CCTA from 1 December 2019 to 30 June 2020. The selection criteria included patients aged ≥18 and ≤80 years; CCTA performed on 64- or higher-detector row computed tomography (CT) scanners; CCTA indicating 30%–90% stenosis in a main coronary artery ≥2.0 mm diameter; and ICA and FFR measurements that were performed within 15 days of the CCTA examination. The exclusion criteria were as follows: lactation, pregnancy, or planned short-term pregnancy; allergy to iodinated contrast medium; adenosine contraindications; prior stent or pacemaker placement; prior coronary artery bypass surgery; artificial heart valve placement; serum creatinine >178 µmol/L; body mass index (BMI) >35 ${\mathrm{kg/m}}^{2}$; heart failure (New York Heart Association grades III or IV); myocardial infarction within one month; poor CCTA imaging quality, diffuse calcification, severe stratification, severe motion artifacts, or other factors leading to failed extraction or modeling of the coronary vascular tree; lesions with aneurysms or myocardial bridges; occlusive lesions; severe tortuosity that would make passing the pressure guide wire through the target vessel difficult; and inability to provide informed consent. Fig. 1 describes the flowchart of patient recruitment.

### 2.2 CCTA Protocol

CCTA was performed in each hospital using a variety of computed tomography scanner platforms with a minimum of 64 detector rows (Aquilion Vision, Toshiba, Otawara, Japan; GE Revolution, GE Healthcare, Milwaukee, Wisconsin; uCT960+, United imaging, Shanghai, China; Somatom Force and Definition Flash, Siemens, Forchheim, Germany). During the collection process, an intravenous infusion of 80–100 mL iodized contrast medium was administered. Image acquisition was performed using either prospective triggering or retrospective gating. Images were acquired of areas including the left ventricle, coronary arteries, and proximal ascending aorta.

### 2.3 Coronary Artery Analysis in CCTA

Two blinded, experienced CT cardiologists analyzed the CCTA images as described in previous studies [9]. The two CT cardiologists analyzed the CCTA images independently, and any disagreements were reconciled through consensus. A three-dimensional (3D) image analysis workstation was used to assess the CCTA images. Coronary artery stenosis was defined as the maximum stenosis identified in all segments within the vascular distribution. Coronary lesions were categorized based on the reduced diameter as a percentage of obstruction into 0%, 1%–29%, 30%–49%, 50%–69%, 70%–90%, subtotally (>90%–99%), or totally (100%) occluded groups. A vessel was classified as uncalcified if the narrower segment was uncalcified. CCTA images were transmitted to an independent central laboratory for calculating the CT-FFR.

### 2.4 CT-FFR Interpretation

CT-FFR calculations were conducted based on regular CCTA data; there was no need to change the data collection protocol, acquire additional images, or administer drugs. The prototype coronary artery physiological function assessment software (Coronary Scope, Shenzhen Yueying Technology Co., Ltd., Shenzhen, Guangdong, China) was installed on a regular workstation of the independent core laboratory (Shenzhen Yueying Technology Co., Ltd., Shenzhen, Guangdong, China). The CT-FFR software was based on NVIDIA’s CUDA-accelerated CFD solver, which divides the solution of the incompressible fluid flow equation into distinct CUDA kernels and suggests a unique implementation that exploits the memory hierarchy of the CUDA programming model. Hence, the CT-FFR software overcomes the highly computationally intensive and time-consuming problem of traditional CT-FFR software.

This CT-FFR algorithm simulates coronary blood flow and patient-specific limit conditions of the hyperemic state established by CFD. The heart rate, diastolic pressure, and systolic pressure of patients are integrated and modified to incorporate the effect of maximal hyperemia to mimic decreases induced by pharmacological stress in microvascular resistance. The CT-FFR was calculated according to the patient’s specific three-dimensional mesh and contour conditions. The patient’s diastolic pressure and systolic pressure of the brachial artery and heart rate were measured before CCTA, and entered into the software. The CT-FFR, at each point of the coronary shaft, was calculated using a three-dimensional color-coded mesh. The CT-FFR is calculated as the mean coronary blood pressure as distal to the pathology as possible divided by the mean arterial blood pressure calculated when simulating maximum congestion. In brief, $F\,F\,R=\frac{D\,i\,s\,t\,a\,l\,C\,o\,r\,o\,n\,a\,r\,y\,P\,r\,e\,s\,s\,u\,r\,e\,\left(P_{d}\right)}{P\,r\,o\,x\,i\,m\,a\,l\,C\,o\,r\,o\,n\,a\,r\,y\,P\,r\,e\,s\,s\,u\,r\,e\,\left(P_{a}\right)}$, where $P_{a},P_{d}$ are calculated by CFD. The analysis was performed by two scientists in the independent, blinded core laboratory. A CT-FFR ≤0.80 or ≤0.75 was considered specific ischemia of the lesion.

The no-new-Net (nnU-Net) deep learning architecture was used to complete automated segmentation of the coronary artery tree. The CT-FFR is based on CUDA-accelerated CFD analysis, which can calculate results with low running time on standard hardware. The nnU-Net is the first segmentation framework to contend with the dataset diversity found in this domain, and is capable of automatically designing and executing a successful network training pipeline for new datasets based on the analysis of existing datasets. Relying on a simple U-Net architecture, nnU-Net can automatically make necessary adjustments to parameters such as preprocessing, batch size, patch size, and inference setting factors that influence several other hyperparameters in the pipeline. Hence, nnU-Net can improve the segmentation accuracy without any manual hyperparameter tuning between different datasets. This process required approximately 5–10 min per case, depending on the quality of CCTA images and the load of atherosclerotic plaque.

### 2.5 ICA Imaging and FFR Performance

Experienced invasive cardiologists performed ICA via a femoral or radial approach. Two experienced invasive cardiologists assessed coronary stenosis on site. Nitroglycerin was administered intracoronary before FFR measurement. A guide cable for pressure monitoring (PressureWire Certus, St. Jude Medical, Inc., Minneapolis, MN, USA) was used. Continuous intravenous (IV) infusion of adenosine (140 µg/kg/min) through the femoral vein. The FFR was obtained automatically as previously described [10]. The gray area of ischemic stenosis recognized by the FFR measurement method was between 0.75 and 0.80.

### 2.6 Statistical Analysis

Categorical variables are presented as frequencies and percentages. Continuous variables are presented as the means ± standard deviations. Either the Student’s *t*-test, Mann–Whitney test, or chi-square test were used to assess differences between groups as appropriate. Receiver operating characteristic (ROC) curve analysis and the area under the ROC curve (AUC) were used to evaluate the performance of the CT-FFR. The Bland–Altman method was used to analyze the systematic difference between the CT-FFR and FFR. All analyses were performed with MedCalc 20.0 (MedCalc Software, Ostend, Belgium). A *p *value of <0.05 was considered statistically significant.

## 3. Results

### 3.1 Patient Characteristics

The study population included a total of 138 patients (age 62.4 ± 9.7 years; 64.5% were men), each undergoing CT-FFR and FFR measurements for stenosis of a single target vessel. Within this group 53 patients exhibited a CT-FFR ≤0.80 and 33 patients had a CT-FFR ≤0.75. The baseline characteristics of the patient population are shown in Table 1. A noteworthy observation was the significant difference in the distribution of hyperlipidemia and sex between patients with a CT-FFR ≤0.75 and CT-FFR >0.75. Furthermore, the average interval between CT-FFR and FFR measurement was just 1.8 days.

**Table 1.** **Patient baseline characteristics**.

Parameter		All (n = 138)	CT-FFR		CT-FFR	
			≤0.80 (n = 53)	>0.80 (n = 85)	≤0.75 (n = 33)	>0.75 (n = 105)
Mean age, yrs		62.4 ± 9.7	60.6 ± 10.1	63.5 ± 9.4	60.8 ± 8.2	62.9 ± 10.2
Male		89 (64.5%)	37 (69.8%)	52 (61.2%)	26 (78.8%)	63 ${\mathrm{(60.0\%)}}^{a}$
BMI, ${\mathrm{kg/m}}^{2}$		24.6 ± 3.0	24.2 ± 2.8	24.8 ± 3.1	24.1 ± 2.3	24.7 ± 3.1
Hypertension		69 (50.0%)	23 (43.4%)	46 (54.1%)	12 (36.4%)	57 (54.3%)
Hyperlipidemia†		29 (21.0%)	14 (26.4%)	15 (17.6%)	11 (33.3%)	18 ${\mathrm{(17.1\%)}}^{a}$
Diabetes mellitus		35 (25.4%)	12 (22.6%)	23 (27.1%)	5 (15.2%)	30 (28.6%)
Smoking						
	Former smokers	14 (10.1%)	7 (13.2%)	7 (8.2%)	5 (15.2%)	9 (8.6%)
	Current smokers	31 (22.5%)	16 (30.2%)	15 (17.6%)	10 (30.3%)	21 (20.0%)
	Never smokers	93 (67.4%)	30 (56.6%)	63 (74.1%)	18 (54.5%)	75 (71.4%)
Cardiovascular history						
	Prior myocardial infarction	2 (1.4%)	0 (0.0%)	2 (2.4%)	0 (0.0%)	2 (1.9%)
	Peripheral vascular diseases	8 (5.8%)	2 (3.8%)	6 (7.1%)	1 (3.0%)	7 (6.7%)
Angina type						
	Typical	99 (71.7%)	40 (75.5%)	59 (69.4%)	27 (81.8%)	72 (68.6%)
	Atypical	39 (28.3%)	13 (24.5%)	26 (30.6%)	6 (18.2%)	33 (31.4%)
Laboratory measures						
	White blood cell count, ×${\mathrm{10}}^{9}$/L	6.3 ± 1.7	6.3 ± 1.6	6.3 ± 1.7	6.7 ± 1.6	6.2 ± 1.7
	Red blood cell count, ×${\mathrm{10}}^{12}$/L	4.5 ± 0.6	4.6 ± 0.5	4.5 ± 0.6	4.6 ± 0.5	4.5 ± 0.6
	Blood platelet count, ×${\mathrm{10}}^{9}$/L	200.6 ± 62.4	206.5 ± 68.2	196.9 ± 58.6	215.2 ± 57.7	196.0 ± 63.5
	Hemoglobin, g/L	138.8 ± 16.3	140.1 ± 15.6	138.1 ± 16.7	141.3 ± 14.9	138.0 ± 16.7
	Creatinine, µmol/L	74.2 ± 18.8	75.1 ± 16.3	73.6 ± 20.3	74.8 ± 16.3	74.0 ± 19.6
	Serum urea, mmol/L	5.5 ± 1.6	5.6 ± 1.5	5.5 ± 1.6	5.3 ± 1.2	5.6 ± 1.6
Interval between CT-FFR and FFR measurement, days		1.8 ± 2.8	2.2 ± 3.2	1.5 ± 2.5	1.7 ± 2.5	1.8 ± 2.9

Data are expressed as the mean ± standard deviation or percentage (%). †Total cholesterol >180 mg/dL or treatment for hypercholesterolemia. Compared with CT-FFR ≤0.75, ^a^*p* < 0.05. BMI, body-mass index; CT-FFR, computed tomography angiography-derived FFR; FFR, fractional flow reserve.

### 3.2 Performance of CCTA Parameters

The CCTA scan parameters are presented in Table 2. The mean tube current and dose length product were 341.0 ± 167.3 mAs and 369.1 ± 275.5 mGy-cm, respectively. There were no significant differences in systolic blood pressure, diastolic blood pressure, heart rate, tube voltage, tube current, or dose length product between groups at a CT-FFR threshold of 0.80 or 0.75.

**Table 2.** **CCTA scan parameters**.

Parameter		All (n = 138)	CT-FFR		CT-FFR	
			≤0.80 (n = 53)	>0.80 (n = 85)	≤0.75 (n = 33)	>0.75 (n = 105)
Vital signs						
	Systolic blood pressure, mmHg	129.0 ± 15.8	128.1 ± 16.4	129.6 ± 15.6	127.8 ± 15.2	129.4 ± 16.1
	Diastolic blood pressure, mmHg	78.2 ± 10.8	80.1 ± 11.9	77.1 ± 10.0	80.8 ± 12.0	77.4 ± 10.3
	Heart rate, beats/min	73.1 ± 11.3	74.4 ± 11.1	72.3 ± 11.3	73.5 ± 11.4	73.0 ± 11.3
Tube voltage						
	70 kV	13 (9.4%)	6 (11.3%)	7 (8.2%)	5 (15.2%)	8 (7.6%)
	80 kV	8 (5.8%)	3 (5.7%)	5 (5.9%)	1 (3.0%)	7 (6.7%)
	100 kV	60 (43.5%)	24 (45.3%)	36 (42.4%)	13 (39.4%)	47 (44.8%)
	120 kV	56 (40.6%)	19 (35.8%)	37 (43.5%)	14 (42.4%)	42 (40.0%)
	140 kV	1 (0.7%)	1 (1.9%)	0 (0.0%)	0 (0.0%)	1 (1.0%)
Tube current (mAs)		341.0 ± 167.3	321.7 ± 178.7	353.1 ± 159.7	348.4 ± 178.8	338.7 ± 164.4
Dose length product (mGy-cm)		369.1 ± 275.5	406.2 ± 343.9	346.1 ± 221.8	370.9 ± 255.1	368.6 ± 282.8

Data are expressed as the mean ± standard deviation or percentage (%). CCTA, coronary computed tomographic angiography; CT-FFR, computed tomography angiography-derived FFR; FFR, fractional flow reserve.

### 3.3 Vessel and Lesion Characteristics

Of the 138 evaluated lesions, two vessels (1.4%) had a left main lesion, 99 vessels (71.7%) had a left anterior descending lesion, 28 vessels (20.3%) had a right coronary artery lesion, and nine vessels (6.5%) had a left circumflex lesion. Thirty-six vessels had 30%–49% stenosis, 64 vessels had 50%–69% stenosis, and 38 vessels had 70%–90% stenosis. Detailed data for vessel and lesion characteristics are shown in Table 3. Lesion-specific ischemia as a function of stenosis category is presented in Table 4.

**Table 3.** **Vessel and lesion characteristics**.

Parameter		All (n = 138)	CT-FFR		CT-FFR	
			≤0.80 (n = 53)	>0.80 (n = 85)	≤0.75 (n = 33)	>0.75 (n = 105)
Target vessel						
	Left main artery	2 (1.4%)	1 (1.9%)	1 (1.2%)	1 (3.0%)	1 (1.0%)
	Left anterior descending	99 (71.7%)	44 (83.0%)	55 (64.7%)	25 (75.8%)	74 (70.5%)
	Right coronary artery	28 (20.3%)	8 (15.1%)	20 (23.5%)	7 (21.2%)	21 (20.0%)
	Left circumflex	9 (6.5%)	0 (0.0%)	9 (10.6%)	0 (0.0%)	9 (8.6%)
Stenosis category						
	30%–49%	36 (26.1%)	5 (9.4%)	31 (36.5%)	4 (12.1%)	32 (30.5%)
	50%–69%	64 (46.4%)	27 (50.9%)	35 (41.2%)	13 (39.4%)	51 (48.6%)
	70%–90%	38 (27.5%)	21 (39.6%)	17 (20.0%)	16 (48.5%)	22 (21.0%)
Plaque features						
	Noncalcified plaque	46 (33.3%)	19 (35.8%)	27 (31.8%)	15 (45.5%)	31 (29.5%)
	Calcified plaque	92 (66.7%)	34 (64.2%)	58 (68.2%)	18 (54.5%)	74 (70.5%)

Data are presented as percentages (%). CT-FFR, computed tomography angiography-derived FFR; FFR, fractional flow reserve.

**Table 4.** **Lesion-specific ischemia as a function of stenosis category**.

Stenosis Category	CT-FFR ≤0.80	CT-FFR ≤0.75	FFR ≤0.80	FFR ≤0.75
30%–49% (n = 36)	5 (17.7%)	4 (11.1%)	7 (19.4%)	6 (16.7%)
50%–69% (n = 64)	27 (42.2%)	13 (20.3%)	25 (39.1%)	16 (25.0%)
70%–90% (n = 38)	21 (55.3%)	26 (68.4%)	21 (55.3%)	19 (50.0%)

Data are presented as percentages (%). CT-FFR, computed tomography angiography-derived FFR; FFR, fractional flow reserve.

### 3.4 CT-FFR and FFR Analysis and Correlation CT-FFR in Identifying Coronary Artery Ischemia

**Fig. 2.**
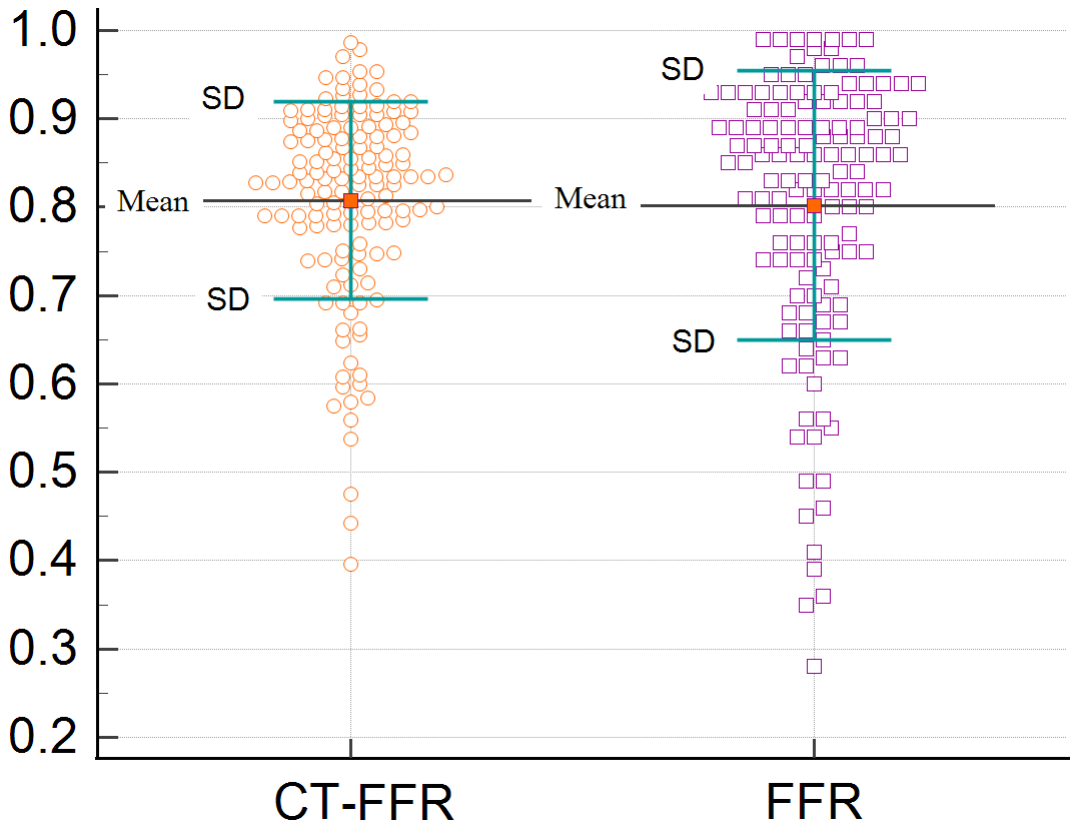
**Distribution of CT-FFR and FFR**. CT-FFR, computed tomography angiography-derived FFR; FFR, fractional flow reserve.

**Fig. 3.**
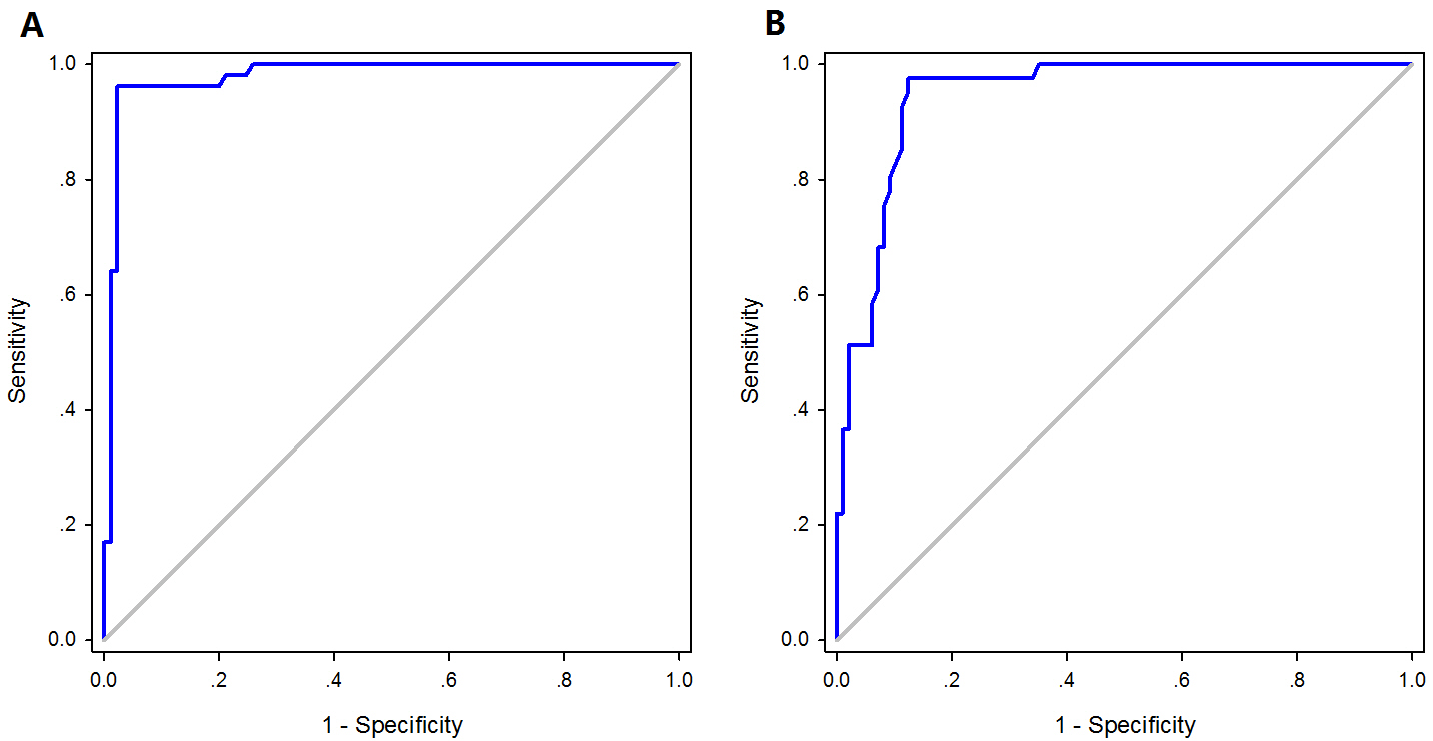
**AUC of CT-FFR ≤0.80 versus FFR ≤0.80 (A) and CT-FFR ≤0.75 versus FFR ≤0.75 (B) in discriminating ischemia**. AUC, area under the receiver operating characteristic curve; CT-FFR, computed tomography angiography-derived FFR; FFR, fractional flow reserve.

**Fig. 4.**
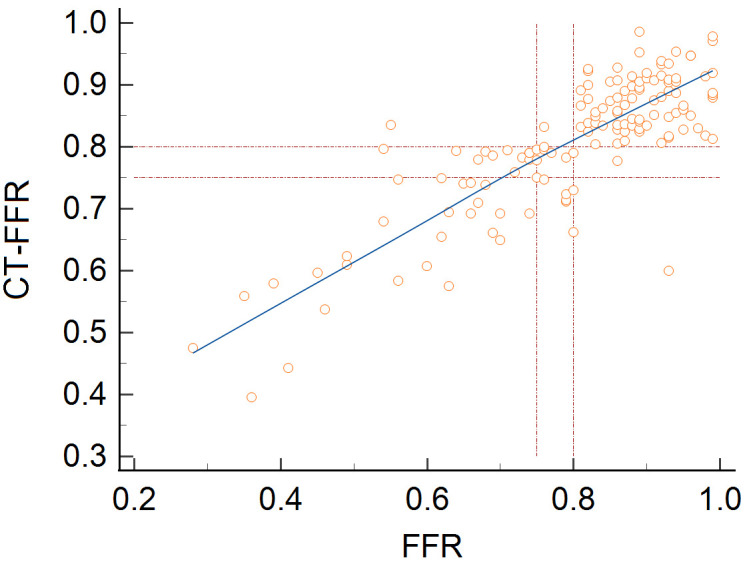
**CT-FFR is related to FFR**. A good Pearson’s correlation coefficient of 0.83 was obtained, *p* < 0.001. CT-FFR, computed tomography angiography-derived FFR; FFR, fractional flow reserve.

**Fig. 5.**
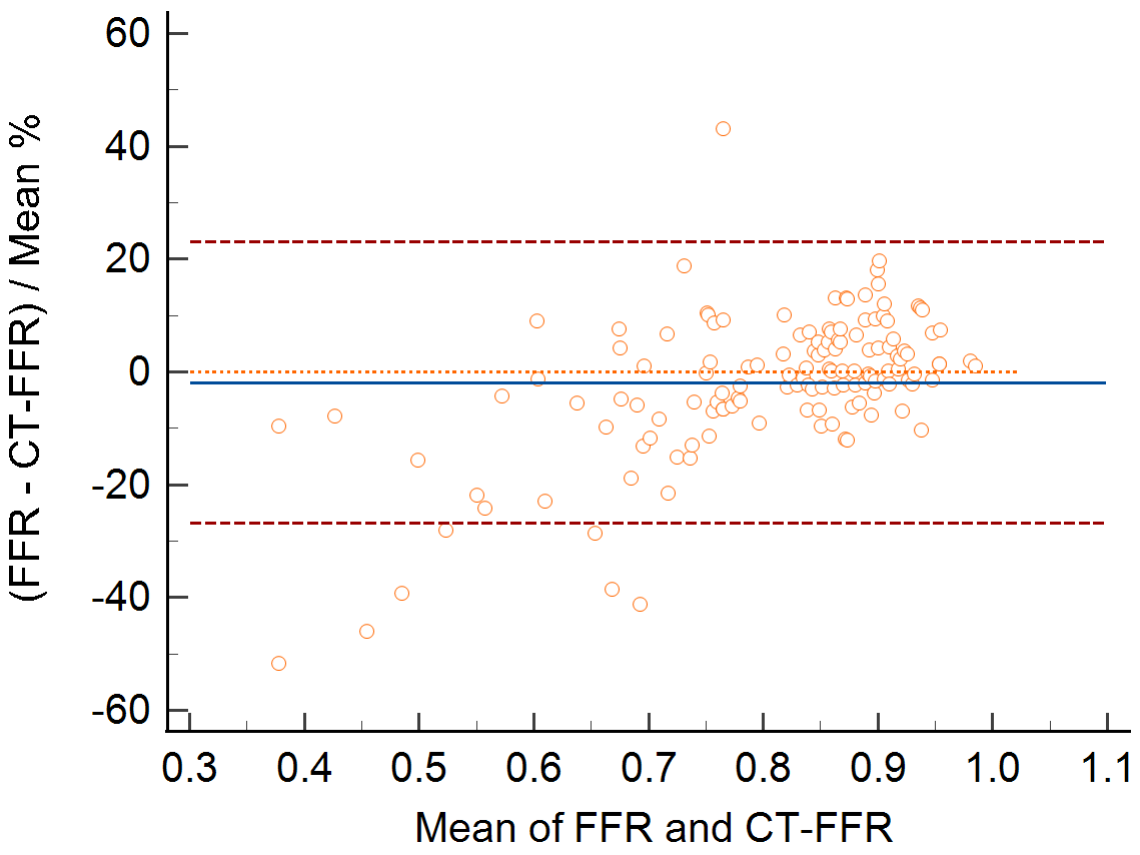
**Bland–Altman plot comparing the FFR and CT-FFR shows no systematic differences (average difference –0.019; 95% agreement limits –0.27 to 0.23)**. CT-FFR, computed tomography angiography-derived FFR; FFR, fractional flow reserve.

The mean CT-FFR was 0.81 ± 0.11, while the mean FFR was 0.80 ± 0.15 (Fig. 2). The diagnostic characteristics of the CT-FFR at both the 0.80 and 0.75 thresholds are presented in Table 5. For the threshold of CT-FFR ≤0.80, the results were as follows: diagnostic accuracy, 97.1%; sensitivity, 96.2%; specificity, 97.7%; positive predictive value (PPV), 96.2%; and negative predictive value (NPV), 97.7%. For the threshold of CT-FFR ≤0.75, the figures were: diagnostic accuracy, 84.1%; sensitivity, 78.8%; specificity, 85.7%; PPV, 63.4%; and NPV, 92.8%. The AUC values were 0.98 (*p* < 0.0001) for CT-FFR ≤0.80 and 0.95 (*p* < 0.0001) for CT-FFR ≤0.75, as seen in Fig. 3. The CT-FFR and FFR had a direct correlation (*p* < 0.001; Fig. 4). There were no significant differences in the Bland–Altman analysis (mean difference –0.019, *p* = 0.085; Fig. 5).

**Table 5.** **CT-FFR metrics in the diagnosis of coronary artery ischemia**.

Measure	CT-FFR ≤0.80 versus FFR ≤0.80	CT-FFR ≤0.75 versus FFR ≤0.75
Accuracy (%)	97.1 (92.7–99.2)	84.1 (76.9–89.7)
Sensitivity (%)	96.2 (87.0–99.5)	78.8 (61.1–91.0)
Specificity (%)	97.7 (91.8–99.7)	85.7 (77.5–91.8)
PPV (%)	96.2 (86.6–99.0)	63.4 (51.2–74.1)
NPV (%)	97.7 (91.4–99.4)	92.8 (86.9–96.1)
Positive likelihood ratio	40.9 (10.4–161.0)	5.5 (3.3–9.1)
Negative likelihood ratio	0.04 (0.01–0.15)	0.25 (0.13–0.48)

CT-FFR, computed tomography angiography-derived FFR; FFR, fractional flow reserve; NPV, negative predictive value; PPV, positive predictive value.

## 4. Discussion

This prospective study revealed that the new CT-FFR deep learning software exhibits a strong direct correlation with FFR and is effective in diagnosing lesion-specific ischemia. Furthermore, we confirmed the efficacy of CT-FFR to detect coronary artery ischemia with stenosis ranging from 30%–90% prior to an ICA referral.

Building on the findings of our study, FFR has emerged as a critical reference for managing coronary artery stenosis, allowing physicians to determine whether revascularization or drug therapy alone is the best course of action. It’s worth noting that the gray area of ischemic stenosis recognized by FFR ranges between 0.75 and 0.80. The well-known DEFER (Deferral versus Performance of Percutaneous Coronary Intervention of Functionally Nonsignificant Coronary Stenosis) and DEFER-DES (Proper Fractional Flow Reserve Criteria for Intermediate Lesions in the Era of Drug-eluting Stent) studies used the lower limit of the gray area (0.75) for decision-making regarding lesion ischemia [11, 12]. Notably, the DEFER randomized controlled study found delayed PCI based on an FFR ≥0.75 was favorable at a 15 years of follow-up. Compared to drug therapy alone, PCI of such functionally insignificant stenosis was not advantageous, and even led to increased myocardial infarction [12]. Moreover, the DEFER-DES study found that unnecessary stent implantation can be avoided by postponing PCI based on an FFR ≥0.75 [11].

The well-known FAME (Fractional Flow Reserve Versus Angiography for Multivessel Evaluation) and FAME 2 trials, which used the upper limit of the gray area (0.80) for FFR, brought interesting insights into the management of coronary artery stenosis [13, 14]. The FAME trial at one year of follow-up and the FAME 2 study at three years of follow-up reported that FFR-guided PCI reduced major cardiovascular events when the FFR was ≤0.80, compared to angiography-guided PCI or drug therapy alone [13, 14]. However, the five-year outcomes of the FAME trial revealed no mortality benefit with invasive FFR-guided PCI for stable CAD [15]. Regardless of whether 0.75 or 0.80 was chosen as the FFR threshold for diagnosing ischemia, the clinical outcome observation for FFR-guided revascularization remained unaffected. This was due to a continuous and independent relationship between clinical outcomes and the FFR for drugs versus revascularization [12]. In this study, we specifically used FFR thresholds of 0.80 and 0.75 to measure the performance of the CT-FFR in detecting coronary ischemia.

CCTA is an established noninvasive modality increasingly used to detect suspicious CAD. However, its inability to assess the hemodynamic effects of lesions and a high false-positive rate result in an overall overestimation of coronary artery stenosis. Even when ICA confirms obstructive coronary lesions diagnosed by CCTA, only a minority lead to coronary ischemia. Therefore, for moderate coronary stenosis determined by CCTA, a functional test is now recommended prior to ICA referral [16].

The need for a validated noninvasive diagnostic method is clear, and the CT-FFR, based on CFD, presents a promising solution. It can accurately identify the hemodynamic effects of lesions and has the potential to significantly reduce unnecessary ICA. A prospective multicenter trial demonstrated the feasibility of CT-FFR, showing a reduction of up to 61% of potential ICA procedures [17]. Furthermore, stable CAD patients with a negative CT-FFR (>0.80) experienced low cardiovascular adverse events at a 12-month follow-up [18]. The advantages of the CT-FFR extend beyond accuracy and include software that aligns with existing CCTA datasets. There is no need to change the data collection protocol, provide additional images, or administer drugs, further streamlining the process.

We investigated novel prototype software for deriving the CT-FFR from CCTA data, which we then compared with the FFR. Previous CT-FFR studies have used a 0.80 threshold to detect lesion-specific ischemia in comparisons with the FFR [19, 20, 21, 22, 23]. Our study revealed that the CT-FFR threshold of 0.80 provided good diagnostic accuracy, sensitivity, and specificity (97.1%, 96.2%, and 97.7% respectively), with an AUC of 0.98, exceeding results from previous studies (Table 6, Ref. [19, 20, 21, 23, 24, 25, 26, 27, 28]). This advancement may be attributed to improvements in the CT-FFR algorithm, the incorporation of deep learning analysis, and unique boundary conditions applied to the new software research prototype. Furthermore, the CT-FFR threshold of 0.75 also exhibited solid diagnostic accuracy, sensitivity, and specificity (84.1%, 78.8%, and 85.7% respectively), with an AUC of 0.95. A direct correlation between CT-FFR and FFR was established (*p* < 0.001) without systematic differences in this study. Cumulatively, these results underscore the CT-FFR’s high diagnostic accuracy in identifying coronary ischemia.

**Table 6.** **Diagnostic Accuracy of CT-FFR software in previous studies at the per-vessel or per-lesion level**.

Study	CT-FFR software	Cut-off value	Accuracy	Sensitivity	Specificity	AUC
Koo *et al*. [19]	HeartFlow V1.0	≤0.80	84.3%	87.9%	82.2%	0.90
Min *et al*. [25]	HeartFlow V1.2	≤0.80	-	80%	61%	-
Nørgaard *et al*. [26]	HeartFlow V1.4	≤0.80	86%	84%	86%	0.93
Renker *et al*. [27]	Siemens cFFR V1.4	≤0.80	-	85%	85%	0.92
Wardziak *et al*. [21]	Siemens cFFR V2.1	≤0.80	74%	76%	72%	0.835
Röther *et al*. [20]	Siemens cFFR V3.0	≤0.80	93%	91%	96%	0.94
Ko *et al*. [28]	Toshiba Medical Systems	≤0.80	83.9%	77.8%	86.8%	0.88
Fujimoto *et al*. [24]	Canon Medical Systems	≤0.80	83.7%	90.9%	78.3%	0.85
Peper *et al*. [23]	IntelliSpace Portal Version 9.0	≤0.80	85.2%	91.2%	81.4%	0.91

CT-FFR, computed tomography angiography-derived FFR; FFR, fractional flow reserve; cFFR, computed fractional flow reserve; AUC, area under the receiver operating characteristic curve.

While the study yielded promising insights, several limitations and unaddressed areas must be acknowledged. First, the relatively low number of samples could influence the robustness of the findings. The inclusion criteria, including 30%–90% coronary artery stenosis, may have introduced selection bias, potentially skewing the results. Furthermore, specific patient conditions, such as previous coronary artery bypass surgery (CABG) and stent implantation, were excluded from the study. This leaves the diagnostic value of the CT-FFR of such patients an open question that requires further examination. Finally, we did not report any clinical outcome observations for CT-FFR–guided revascularization, leaving an area for future exploration.

## 5. Conclusions

In this prospective trial, we utilized novel CT-FFR software to analyze CCTA data, comparing its findings with the established FFR. The key results include a strong direct correlation between the CT-FFR and FFR, along with high diagnostic performance for lesion-specific ischemia, particularly within a stenosis rate of 30%–90%. This study highlights the accuracy and clinical value of the CT-FFR, particularly when leveraging deep learning analysis. However, the findings are subject to certain limitations, notably the specificity of the inclusion and exclusion criteria, making them applicable only to specific patients and types of coronary stenosis. Consequently, the general applicability of the current conclusions require further study. Additionally, future studies should evaluate the novel CT-FFR software’s impact on CAD patients’ prognosis and compare it with other CT-FFR software solutions.

## Data Availability

The datasets used and/or analyzed during the current study are available from the corresponding author on reasonable request.

## References

[1] Liga R, Vontobel J, Rovai D, Marinelli M, Caselli C, Pietila M (2016). Multicentre multi-device hybrid imaging study of coronary artery disease: results from the EValuation of INtegrated Cardiac Imaging for the Detection and Characterization of Ischaemic Heart Disease (EVINCI) hybrid imaging population. *European Heart Journal Cardiovascular Imaging*.

[2] Wong CCY, Ng ACC, Ada C, Chow V, Fearon WF, Ng MKC (2021). A real-world comparison of outcomes between fractional flow reserve-guided versus angiography-guided percutaneous coronary intervention. *PLoS ONE*.

[3] Montalescot G, Sechtem U, Achenbach S, Andreotti F, Arden C, Budaj A (2013). 2013 ESC guidelines on the management of stable coronary artery disease: the Task Force on the management of stable coronary artery disease of the European Society of Cardiology. *European Heart Journal*.

[4] Kim YJ, Yong HS, Kim SM, Kim JA, Yang DH, Hong YJ (2015). Korean Guidelines for the Appropriate Use of Cardiac CT. *Korean Journal of Radiology*.

[5] Celeng C, Leiner T, Maurovich-Horvat P, Merkely B, de Jong P, Dankbaar JW (2019). Anatomical and Functional Computed Tomography for Diagnosing Hemodynamically Significant Coronary Artery Disease: A Meta-Analysis. *JACC: Cardiovascular Imaging*.

[6] Zhuang B, Wang S, Zhao S, Lu M (2020). Computed tomography angiography-derived fractional flow reserve (CT-FFR) for the detection of myocardial ischemia with invasive fractional flow reserve as reference: systematic review and meta-analysis. *European Radiology*.

[7] Coenen A, Kim Y, Kruk M, Tesche C, De Geer J, Kurata A (2018). Diagnostic Accuracy of a Machine-Learning Approach to Coronary Computed Tomographic Angiography-Based Fractional Flow Reserve: Result From the MACHINE Consortium. *Circulation. Cardiovascular Imaging*.

[8] Qiao HY, Tang CX, Schoepf UJ, Tesche C, Bayer RR, Giovagnoli DA (2020). Impact of machine learning–based coronary computed tomography angiography fractional flow reserve on treatment decisions and clinical outcomes in patients with suspected coronary artery disease. *European Radiology*.

[9] Raff GL, Abidov A, Achenbach S, Berman DS, Boxt LM, Budoff MJ (2009). SCCT guidelines for the interpretation and reporting of coronary computed tomographic angiography. *Journal of Cardiovascular Computed Tomography*.

[10] Pijls NHJ, Kern MJ, Yock PG, De Bruyne B (2000). Practice and potential pitfalls of coronary pressure measurement. *Catheterization and Cardiovascular Interventions*.

[11] Park SH, Jeon KH, Lee JM, Nam CW, Doh JH, Lee BK (2015). Long-Term Clinical Outcomes of Fractional Flow Reserve-Guided Versus Routine Drug-Eluting Stent Implantation in Patients With Intermediate Coronary Stenosis: Five-Year Clinical Outcomes of DEFER-DES Trial. *Circulation: Cardiovascular Interventions*.

[12] Zimmermann FM, Ferrara A, Johnson NP, van Nunen LX, Escaned J, Albertsson P (2015). Deferral vs. performance of percutaneous coronary intervention of functionally non-significant coronary stenosis: 15-year follow-up of the DEFER trial. *European Heart Journal*.

[13] Tonino PAL, De Bruyne B, Pijls NHJ, Siebert U, Ikeno F, van’t Veer M (2009). Fractional Flow Reserve versus Angiography for Guiding Percutaneous Coronary Intervention. *New England Journal of Medicine*.

[14] Fearon WF, Nishi T, De Bruyne B, Boothroyd DB, Barbato E, Tonino P (2018). Clinical Outcomes and Cost-Effectiveness of Fractional Flow Reserve-Guided Percutaneous Coronary Intervention in Patients With Stable Coronary Artery Disease: Three-Year Follow-Up of the FAME 2 Trial (Fractional Flow Reserve Versus Angiography for Multivessel Evaluation). *Circulation*.

[15] Xaplanteris P, Fournier S, Pijls NHJ, Fearon WF, Barbato E, Tonino PAL (2018). Five-Year Outcomes with PCI Guided by Fractional Flow Reserve. *New England Journal of Medicine*.

[16] Timmis A, Roobottom CA (2017). National Institute for Health and Care Excellence updates the stable chest pain guideline with radical changes to the diagnostic paradigm. *Heart*.

[17] Douglas PS, Pontone G, Hlatky MA, Patel MR, Norgaard BL, Byrne RA (2015). Clinical outcomes of fractional flow reserve by computed tomographic angiography-guided diagnostic strategies vs. usual care in patients with suspected coronary artery disease: the prospective longitudinal trial of FFR(CT): outcome and resource impacts study. *European Heart Journal*.

[18] Nørgaard BL, Gaur S, Fairbairn TA, Douglas PS, Jensen JM, Patel MR (2022). Prognostic value of coronary computed tomography angiographic derived fractional flow reserve: a systematic review and meta-analysis. *Heart*.

[19] Koo BK, Erglis A, Doh JH, Daniels DV, Jegere S, Kim HS (2011). Diagnosis of ischemia-causing coronary stenoses by noninvasive fractional flow reserve computed from coronary computed tomographic angiograms. Results from the prospective multicenter DISCOVER-FLOW (Diagnosis of Ischemia-Causing Stenoses Obtained Via Noninvasive Fractional Flow Reserve) study. *Journal of the American College of Cardiology*.

[20] Röther J, Moshage M, Dey D, Schwemmer C, Tröbs M, Blachutzik F (2018). Comparison of invasively measured FFR with FFR derived from coronary CT angiography for detection of lesion-specific ischemia: Results from a PC-based prototype algorithm. *Journal of Cardiovascular Computed Tomography*.

[21] Wardziak Ł, Kruk M, Pleban W, Demkow M, Rużyłło W, Dzielińska Z (2019). Coronary CTA enhanced with CTA based FFR analysis provides higher diagnostic value than invasive coronary angiography in patients with intermediate coronary stenosis. *Journal of Cardiovascular Computed Tomography*.

[22] Chung J, Lee KE, Nam C, Doh J, Kim HI, Kwon S (2017). Diagnostic Performance of a Novel Method for Fractional Flow Reserve Computed from Noninvasive Computed Tomography Angiography (NOVEL-FLOW Study). *The American Journal of Cardiology*.

[23] Peper J, Schaap J, Rensing BJWM, Kelder JC, Swaans MJ (2022). Diagnostic accuracy of on-site coronary computed tomography-derived fractional flow reserve in the diagnosis of stable coronary artery disease. *Netherlands Heart Journal*.

[24] Fujimoto S, Kawasaki T, Kumamaru KK, Kawaguchi Y, Dohi T, Okonogi T (2019). Diagnostic performance of on-site computed CT-fractional flow reserve based on fluid structure interactions: comparison with invasive fractional flow reserve and instantaneous wave-free ratio. *European Heart Journal Cardiovascular Imaging*.

[25] Min JK, Leipsic J, Pencina MJ, Berman DS, Koo B, van Mieghem C (2012). Diagnostic Accuracy of Fractional Flow Reserve from Anatomic CT Angiography. *JAMA*.

[26] Nørgaard BL, Leipsic J, Gaur S, Seneviratne S, Ko BS, Ito H (2014). Diagnostic performance of noninvasive fractional flow reserve derived from coronary computed tomography angiography in suspected coronary artery disease: the NXT trial (Analysis of Coronary Blood Flow Using CT Angiography: Next Steps). *Journal of the American College of Cardiology*.

[27] Renker M, Schoepf UJ, Wang R, Meinel FG, Rier JD, Bayer RR (2014). Comparison of Diagnostic Value of a Novel Noninvasive Coronary Computed Tomography Angiography Method Versus Standard Coronary Angiography for Assessing Fractional Flow Reserve. *The American Journal of Cardiology*.

[28] Ko BS, Cameron JD, Munnur RK, Wong DTL, Fujisawa Y, Sakaguchi T (2017). Noninvasive CT-Derived FFR Based on Structural and Fluid Analysis: A Comparison With Invasive FFR for Detection of Functionally Significant Stenosis. *JACC Cardiovasc Imaging*.

